# A review of water electrolysis–based systems for hydrogen production using hybrid/solar/wind energy systems

**DOI:** 10.1007/s11356-022-23323-y

**Published:** 2022-10-25

**Authors:** Mohamed Nasser, Tamer F. Megahed, Shinichi Ookawara, Hamdy Hassan

**Affiliations:** 1 Energy Resources Engineering Department, Egypt-Japan University of Science and Technology (E-JUST), Alexandria, Egypt; 2 Mechanical Power Engineering Department, Faculty of Engineering, Zagazig University, Zagazig, Egypt; 3 Electrical Power Engineering Department, Egypt-Japan University of Science and Technology (E-JUST), Alexandria, Egypt; 4 Electrical Engineering Department, Mansoura University, Mansoura, Egypt; 5 Tokyo Institute of Technology, Tokyo, Japan; 6 Mechanical Power Engineering Department, Faculty of Engineering, Assiut University, Asyut, Egypt

**Keywords:** Clean hydrogen, Sustainable hydrogen production, Hydrogen economy, Renewable energy, Low/high-temperature electrolyzers, Multi-generation system

## Abstract

Hydrogen energy, as clean and efficient energy, is considered significant support for the construction of a sustainable society in the face of global climate change and the looming energy revolution. Hydrogen is one of the most important chemical substances on earth and can be obtained through various techniques using renewable and nonrenewable energy sources. However, the necessity for a gradual transition to renewable energy sources significantly hampers efforts to identify and implement green hydrogen production paths. Therefore, this paper’s objective is to provide a technological review of the systems of hydrogen production from solar and wind energy utilizing several types of water electrolyzers. The current paper starts with a short brief about the different production techniques. A detailed comparison between water electrolyzer types and a complete illustration of hydrogen production techniques using solar and wind are presented with examples, after which an economic assessment of green hydrogen production by comparing the costs of the discussed renewable sources with other production methods. Finally, the challenges that face the mentioned production methods are illuminated in the current review.

## Introduction

Due to increased world populations, the extensive exploration and use of fossil fuels have led to several environmental issues harming human health and life (Khan et al. 2022). Therefore, current primary concerns have included ways to provide a cost-effective, dependable, and environmentally friendly primary energy source with as low carbon emissions as possible. Furthermore, this energy source should be sustainable and accessible in every region (Chien et al. 2021) (Eldesoukey and Hassan 2019). Hence, there is an urgent need to find and use an alternative clean energy source that is renewable and safe enough to replace nonrenewable sources. However, abundant difficulties are inherent in renewable energy power plants (Zhang et al. 2006; Abdelshafy et al. 2018). For example, these plants are installed in arid regions and need a storage system due to the intermittent nature of renewable sources (Singla et al. 2021).

Based on these issues, hydrogen, which is considered an alternative energy carrier, is proposed to play a significant role in future energy because it can be stored and transported and has a high calorific combustion value, making it suitable to replace fossil fuels (Saxena et al. 2008). Its eco-friendly production process also accounts for one of its key features on the road to a better environment and the success of sustainable development (Joshi et al. 2010). Moreover, hydrogen can be directly applied to fuel cells to produce electricity without any toxic emissions but with an energy yield of about 122 KJ/g, which is 2.75 times greater than hydrocarbon fuels (Fan et al. 2021). Table 1 shows the thermophysical properties of hydrogen.

**Table 1:** Hydrogen thermophysical properties (Dincer and Zamfirescu 2011; Saxena et al. 2008)

Property	Value
Hydrogen	H_2_
Density at STP	0.084 Kg/m^3^
LHV	120 KJ/g
HHV	141.8 KJ/g
Melting point	14.01 K
Normal boiling point	20.3 K
Critical temperature	32.97 K
Critical pressure	12.9 bar
Flammability limits in the air (vol%)	4.1–75%
Autoignition temperature	858 K
Adiabatic flame temperature	2400 k
Flame speed	2.75 m/s
LHV, lower heating value; HHV, higher heating value	

**Fig. 1:**
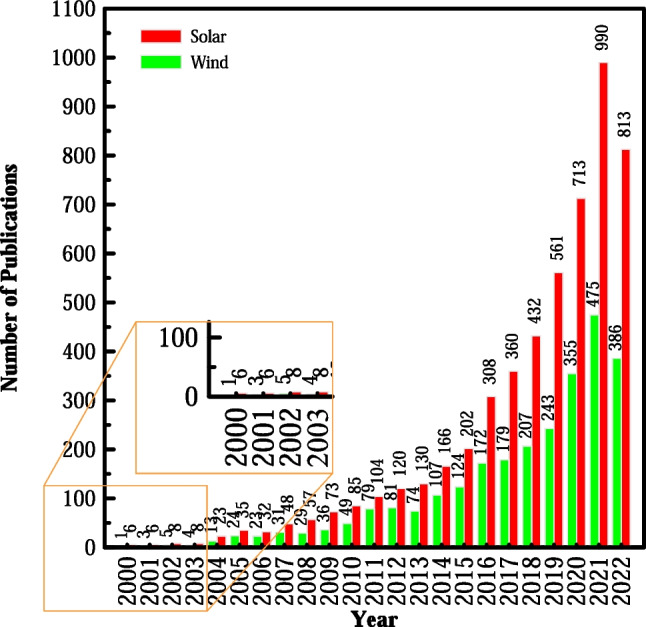
Number of published articles on hydrogen production using solar and wind energy (Elsevier 2022)

Therefore, this paper provides a general overview of the hydrogen production techniques according to feedstock type and energy source, focusing on hydrogen production systems from water electrolysis using solar and wind energy. Furthermore, a detailed comparison between different electrolyzer types was conducted, focusing on their advantages and disadvantages. In the final section, an economic assessment of the understudied production system is then illustrated to show the hydrogen production costs and challenges that face each technique. From the details of this paper, we propose to help researchers develop a good understanding of clean hydrogen production techniques through water electrolysis using wind and solar since these sources have been on an upward curve since 2000, as illustrated in Fig. 1. This figure demonstrates the number of published articles per year, which are in ascending trend because of the increased interest in alternative energy sources. It is noted that solar energy is superior to wind power in terms of hydrogen production.

Alternatively, although solar energy is superior to wind power in hydrogen production, electrolysis generally has significant downsides, such as when using platinum-based electrocatalytic metals or due to high energy demands and observed corrosion at the cathode. Hence, several patent innovations have been primarily proposed concerning the search for electrode material enhancements (Martinez-Burgos et al. 2021). To this end, the green hydrogen production process has accounted for increasing patents since 2005, with the number of patents in 2005 being 55 but increasing to 375 inventions in 2020 (an increase of 588%). Notably, Japan and the United States have a considerable lead in the number of innovations (IRENA 2022).

## Hydrogen production technologies

**Fig. 2:**
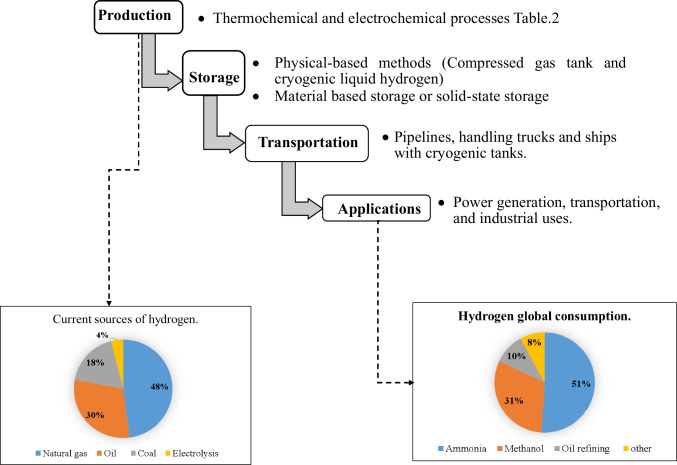
Stages of the hydrogen industry (Arregi et al. 2018; Abohamzeh et al. 2021)

The hydrogen industry is divided into four major parts: production, storage, transportation, and use (Fig. 2). As shown in Fig. 2, while natural gas is the primary source of hydrogen production, ammonia manufacturing is the most hydrogen-consuming industry. Furthermore, hydrogen transportation from the production site to the consumption site is a vital process in the hydrogen production economies because it can increase production costs, influencing its affordability. Hydrogen storage is considered an urgent and challenging stage because it helps develop safe, reliable, efficient, and adequate storage mechanisms (Zhang et al. 2016). Therefore, hydrogen production processes based on feedstocks have also been proposed. Table 2 briefly explains the hydrogen production processes, focusing on water electrolysis.

**Table 2:** Summary of the hydrogen production techniques according to feedstock types (Hacker et al. 2000; Steinfeld 2005; Kovács et al. 2006; Gupta 2009; Holladay et al. 2009; Zhang et al. 2016; Buttler and Spliethoff 2018; David et al. 2019; El-Emam and Özcan 2019; Hassan et al. 2021)

Feedstocks	Energy	Production process	Description
Water	Solar	Photolysis	• Sunlight is used directly to produce H_2_ from water
			• Long-term technology with low production efficiency
	Electricity^a^	Electrolysis	• Decomposition of water to H_2_ and O_2_ due to the electric current passing through the electrolyzer
			• Commercial technology
			• H_2_ production efficiency is 60–90%^**b**^
	Thermal^**c**^	Thermochemical water splitting (thermolysis)	• Decomposition of water due to heat energy (~2500°C)
			• Efficiency is about 50%
	Electricity^**a**^ and solar	Photo-electrochemical water splitting	• Water decomposition is due to sunlight and electricity
			• Efficiency is about 12.4%
Biomass	Thermal^**c**^	Gasification	• Solid fuel reacts with O_2_ and/or steam to produce H_2_ and CO_2_
			• Efficiency is about 35–50%^**d**^
	Biochemical	Dark fermentation	• Primarily anaerobic bacteria carry out the reaction, sometimes algae, that converts carbohydrate-rich matter into H_2_, CO_2_, and other products
			• Efficiency is about 60–80%
	Solar	Photo fermentation	• Solar to hydrogen via organic materials occurs in light
			• Efficiency is about 0.1%
	Electricity^**a**^	Microbial electrolysis cell	• The use of electrohydrogenesis converts biodegradable material into hydrogen
			• Efficiency is about 78%
Hydrocarbons	Thermal^**c**^	Steam reforming	• A steam reaction with liquid or gas fuel at a high temperature
			• Efficiency is about 70–85%
		Partial oxidation	• The reaction of hydrocarbons with O_2_ at high temperatures
			• The reaction of methane with O_2_
			• Efficiency is about 60–75%
		Autothermal reforming	• The reaction of O_2_ and steam with hydrocarbons
			• Efficiency is about 60–75%
		Thermal decomposition (pyrolysis)	• Hydrocarbons thermally break down into hydrogen and carbon when heated to a high temperature
			• Efficiency is about 60%
		Steam–iron process	• Steam–iron hydrogen synthesis is one of the earliest commercial processes
			• Coal is consumed cyclically by water cleavage. Coal is gasified to carbon monoxide and hydrogen
	Electricity^**a**^	Plasma reforming	• The same as the conventional reforming process
			• Efficiency is about 9–85%
Other	Thermal^**c**^	Ammonia reforming	• Use portable power applications
			• Near-term technology
		Aqueous phase reforming	• Use carbohydrates as a feedstock
			• It occurs under 25–30 MPa and 220–270°C
			• Efficiency is about 35–55%

^**a**^Electricity is produced from renewable energy sources, grid, or energy recovery

^**b**^The electrolyzer efficiency is based on HHV of hydrogen (El-Emam and Özcan 2019)

^**c**^Thermal energy can be produced from several energy sources: solar, geothermal, and nuclear

^d^The thermal efficiency is based on a higher heating value

## Water electrolyzer

Two essential components of a green hydrogen system are its renewable energy source and the use of a water electrolyzer. During water electrolysis, water decomposes into hydrogen and oxygen under electricity using an electrolyzer. Therefore, due to its intermittency, this electrolyzer has been proposed as the most feasible and commercial method for hydrogen production and energy storage when coupled with renewable energy. Based on these facts, the most common electrolyzers are the proton exchange membrane electrolyzer (PEM), alkaline water electrolyzer (AWE), alkaline anion exchange membrane (AEM), and solid oxide electrolyzer (SOE) (Chi and Yu 2018; Lim and Kim 2022). Table 3 demonstrates the main points distinguishing each electrolyzer to show the difference between each type. The main benefits of renewable energy/water electrolyzers are as follows: (1) the possible reduction or elimination of transportation and storage costs because they can be used as stand-alone systems for end-user sites, (2) their compactness and the possibility of high hydrogen production against photo-electrochemical, (3) lack of electricity infrastructural needs in arid regions, and (4) their commercially available nature (Nasser et al. 2022a).

**Table 3:**
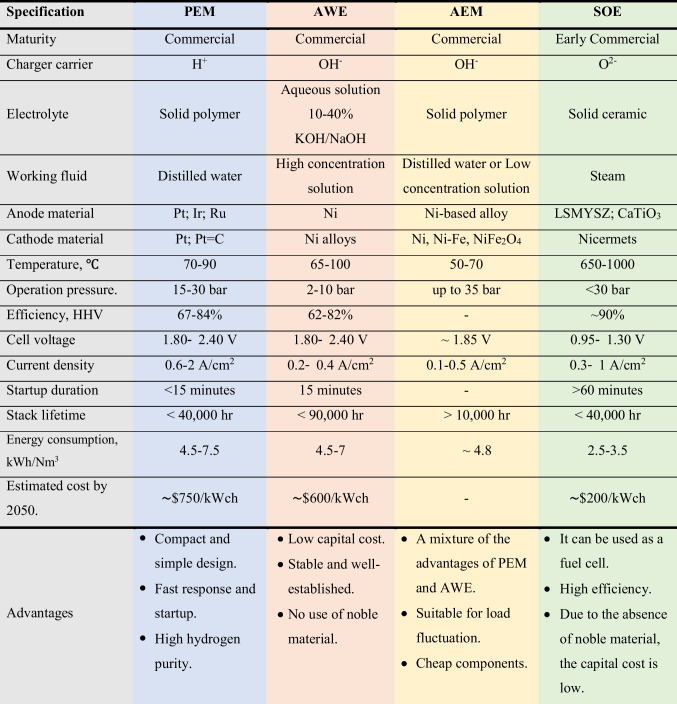

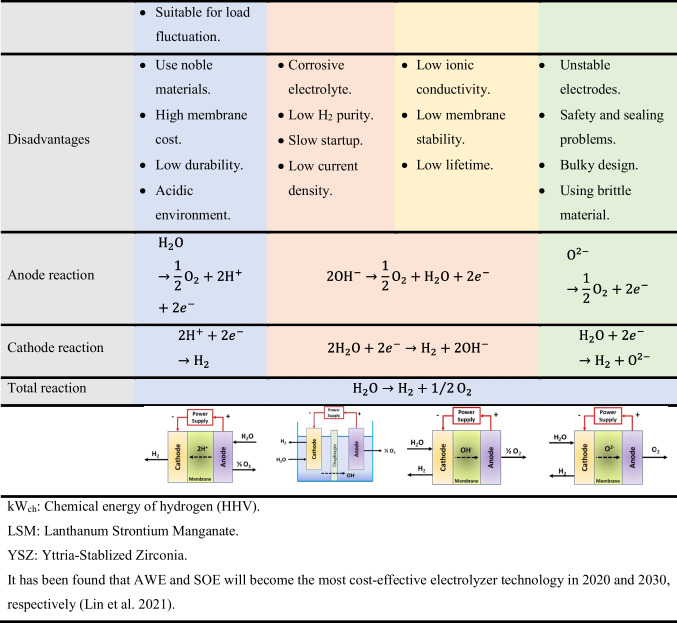
Comparison between different types of water electrolyzers (Dincer and Acar 2014; Chi and Yu 2018; El-Emam and Özcan 2019; Hosseini and Wahid 2020; Burton et al. 2021; Motealleh et al. 2021; Li and Baek 2021)

kWch: Chemical energy of hydrogen (HHV).

LSM: Lanthanum Strontium Manganate.

YSZ: yYttria-Stabilized Zirconia.

It has been found that AWE and SOE will become the most cost-effective electrolyzer technology in 2020 and 2030, respectively (Lin et al. 2021).

Electrolyzer stacks comprise many connected cells, categorized into monopolar and bipolar types. While the bipolar design connects the cells in series, the monopolar cells are connected electrically and geometrically in parallel. Consequently, electric wiring is less in the bipolar one due to its compactness, enhancing its efficiency. However, this type’s main drawback is its high cost due to its complex design compared with monopolar stacks (Zhang et al. 2016).

### Proton exchange membrane electrolyzer (PEM)

A PEM electrolyzer was firstly introduced in the 1960s by General Electric (Buttler and Spliethoff 2018). The fundamental components of PEM are its anode, cathode, and electrolyte (Table 3), while the most common materials for anode and cathode are platinum, iridium, ruthenium, and platinum on carbon. The Chemours Company FC, LLC, with trademark Nafion and FUMATECH BWT GmbH with trademark Fumapem are the typical suppliers for the PEM membrane. Alternatively, electrolyte materials are responsible for the high conductivity of protons, low gas crossover, compact design of an electrolyzer, and high operation pressure (15–30 bar at 50–90°C) (Carmo et al. 2013).

The significant PEM advantages are that it can perfectly deal with load fluctuation due to its rapid response, with its produced hydrogen purity up to 99.999% (Buttler and Spliethoff 2018). In contrast, the main disadvantage, until now, is its high cost due to the noble material used inside the electrolyzer (Bhandari et al. 2014). Table 3 shows PEM’s operation principle and main parameters.

### Alkaline water electrolyzer (AWE)

AWE is the most mature technology among the other types. It is reliable and safe and can be maintained in a large-scale unit (Yan and Hino 2018). This electrolyzer is composed of two electrodes submerged in a liquid electrolyte water solution, usually 20–40% sodium hydroxide (NaOH) or potassium hydroxide (KOH) (Zhang et al. 2016). A diaphragm separates these electrodes in the solution, allowing water molecules and hydroxide ions to pass through. The diaphragm also separates H_2_ and O_2_ for safety and purity aspects (Carmo et al. 2013; El-Emam and Özcan 2019) (Table 3). Consequently, the purity of the produced hydrogen is 99.5 to 99.9% and can be increased up to 99.999% by catalytic gas purification processes (Buttler and Spliethoff 2018).

**Fig. 3:**
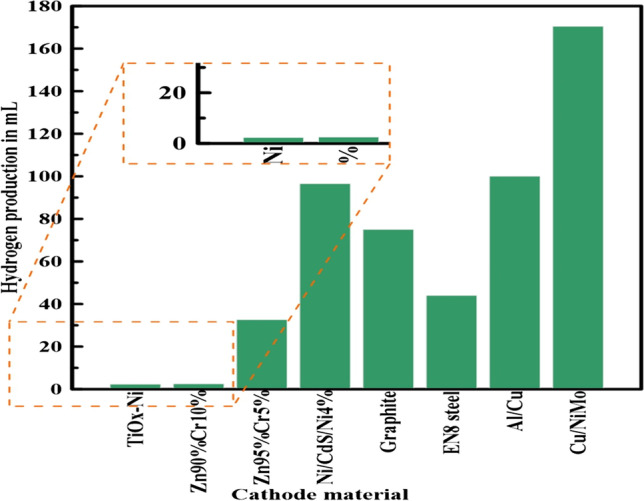
Hydrogen production from AWE at different cathode materials (Mert et al. 2019)

Notably, AWE performance is influenced by the diaphragm, anode, and cathode material type and thickness. As an example, Fig. 3 demonstrates the effect of cathode material on hydrogen production (Mert et al. 2019). Investigations revealed that while the Cu/NiMo cathode has the highest hydrogen production, the Cu cathode has the lowest. Moreover, although an electrolyte’s temperature does not affect hydrogen production and lowers the required power (Rahim et al. 2015), an electrolyte solution’s concentration affects the output. Therefore, the main difference between PEM and AWE is the electrolyte type since PEMs use a solid polymer membrane electrolyte, but AWEs use a corrosive liquid electrolyte.

### Alkaline anion exchange membrane (AEM)

AEM has recently been designed as an alternative to traditional water electrolyzers. Interestingly, AWE and PEM are combined in AEM to address some of the drawbacks of the first and second electrolyzer types. Hence, it combines a low-concentration alkaline solution as opposed to a 20–40% KOH or NaOH aqueous solution with a solid electrolyte (polymeric) membrane (e.g., Mg-Al LDH) (Cho et al. 2018; Li and Baek 2021). Furthermore, the anode in an AEM is manufactured from Ni-based (e.g., Ni foams) or titanium materials, and the cathode comprises Ni, Ni-Fe, and NiFe_2_O_4_ (Faid et al. 2018; Chi and Yu 2018; Li and Baek 2021). Table 3 shows the reaction inside AEM and its working principle.

### Solid oxide electrolyzer (SOE)

Although SOE operates at a high temperature, the electricity required to drive its electrolysis process at such a high temperature is significantly reduced compared to low-temperature electrolysis. Therefore, the system’s efficiency is improved because it uses inexpensive thermal energy or waste heat. Furthermore, while the cathode material is made from a 50/50 wt% mixture of lanthanum strontium manganite and yttrium-stabilized zirconia, the anode and electrolyte materials are cermets and ceramic, respectively (El-Emam and Özcan 2019). However, SOE must undergo further research and development to provide better catalyst and electrode materials (El-Emam and Özcan 2019).

Its hydrogen production process is described as follows: First, steam at the cathode side is reduced to hydrogen according to the cathode reaction, and then, the oxide anions generated on the cathode side are the path through which solid electrolytes form oxygen on the anode side. Table 3 summarizes SOE’s characteristics, specifications, advantages, and disadvantages.

### Challenges of water electrolysis

The primary goal of commercializing hydrogen generation using electrolysis is to reduce investment and operational expenses (Younas et al. 2022). While it is possible to build renewable water electrolysis systems using currently available technologies, the system’s costs are unlikely to decrease soon without a dramatic breakthrough in solar and wind technology. Other issues, such as the intermittent nature of energy sources, water consumption rates, and their efficiencies, also need to be addressed. Therefore, this electrolysis method is considered less attractive, considering its hydrogen production cost.

## Green electricity production systems

Solar and wind energy produces sufficient electricity to drive electrolyzers for hydrogen storage or direct use during production. Typical examples of solar energy are photovoltaic (PV) and concentrated solar power (CSP) systems (Soliman et al. 2019). However, wind turbines used to convert wind to power are an example of wind energy. Although PV panels and wind turbines are directly coupled with electrolyzers, CSP is first associated with a power cycle for electricity production before connecting them to electrolyzers. Therefore, the need for an AC/DC or DC/DC converter is mandatory in electrolyzer load adjustments.

**Fig. 4:**
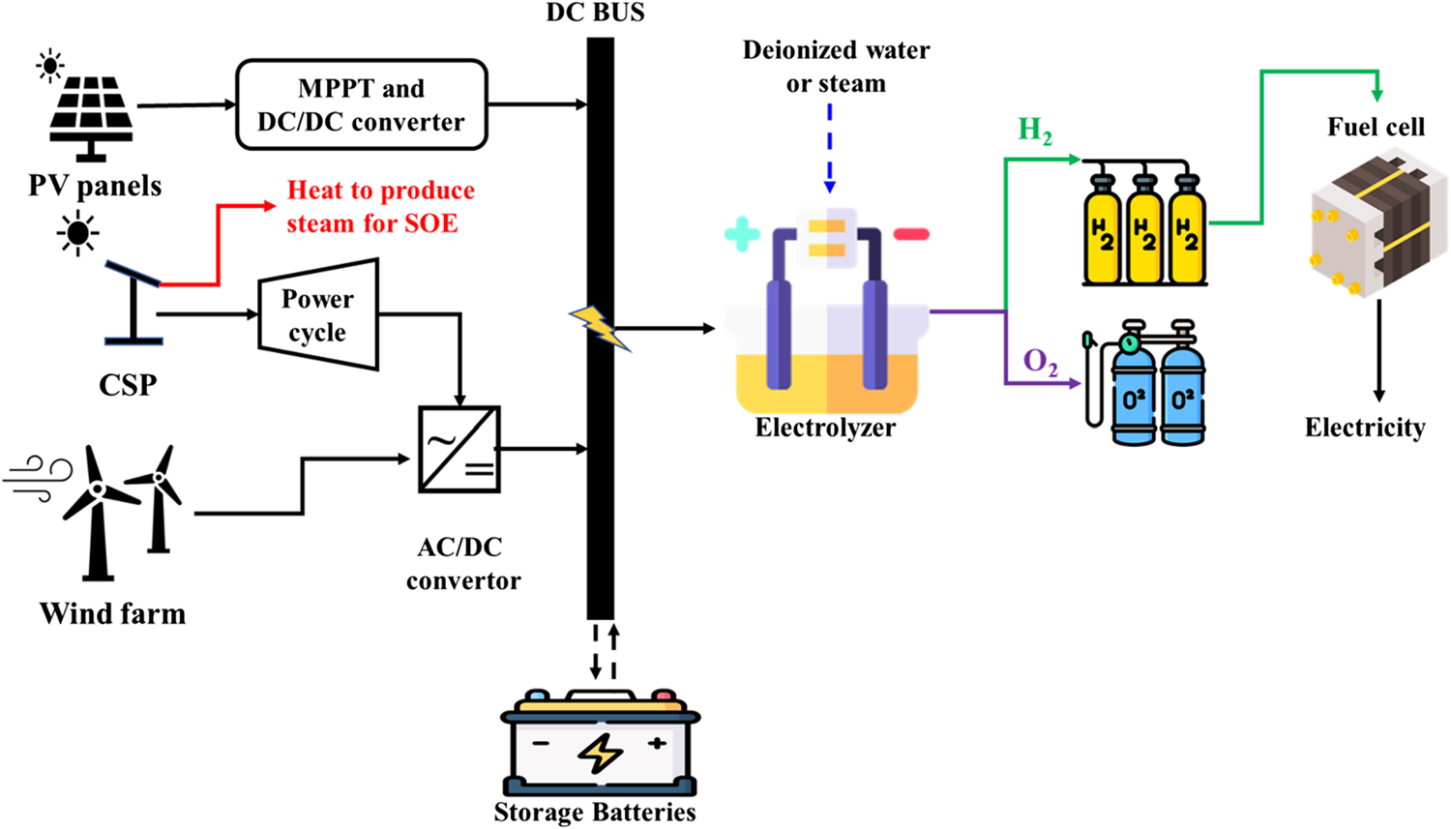
The schematic diagram for solar/wind hydrogen production systems

Green production offers an ideal solution to provide remote areas with power due to the high cost of power transmission (Singla et al. 2021). Therefore, excess energy from renewable sources has been used to operate electrolyzers for hydrogen production. Hydrogen can also be used in fuel cells to produce electricity during the night or intermittency. Fig. 4 presents the basic concept of a solar/wind hydrogen production system.

In a PV/hydrogen production (PV/H_2_) system, PV panels are linked to an electrolyzer through a power-conditioning unit containing a maximum power point tracking (MPPT) system and a DC/DC converter. This unit is applied to maximize the output from panels and adjust the electrolyzer input power (Haider et al. 2021; Nasser et al. 2022b). However, in the case of excess electricity from PV systems, a battery system is adopted as energy storage. Therefore, the main benefits of the PV/H_2_ system over other systems are the use of a DC electricity output and the absence of moving parts, which leads to minor maintenance. Contrastively, in the CSP/hydrogen production (CSP/H_2_) system, solar radiation heat is divided into two portions: the first is used in power cycles (e.g., organic Rankine cycle (ORC)) to generate electricity that drives the electrolyzer, and the second converts water to steam by employing SOE, as mentioned in Fig. 4 (Chadegani et al. 2018). Based on this principle, the system may have thermal storage to ensure continuous production at night. A previous study compared PV/H_2_ and CSP/H_2_ under the same conditions to investigate their system performances (Joshi et al. 2011). The results revealed that the CSP/H_2_ system performed better than PV/H_2_.

Comparatively, the wind/hydrogen production (wind/H_2_) system is more like a PV/H_2_ system but needs an AC/DC converter to drive the electrolyzer. Although wind energy is available throughout the day in contrast to solar energy, this system has a significant weakness of wind’s unpredictable nature. Fig. 4 illustrates the essential components of this system. Interestingly, a combination of these energy sources can be applied to enhance a system’s efficiency and provide a multi-generation cycle. Therefore, this possibility is discussed in detail in the current study.

### The PV/H_2_ system

The PV/H_2_ system is a promising technique for green hydrogen production due to its economically competitive, commercially viable, and sustainable structure (Bhattacharyya et al. 2017). Therefore, this system has been investigated under different climatic conditions with and without a solar tracking system (Bilgen 2001). Notably, a study indicated that although the system with solar tracking had higher performance, its capital cost was raised. Moreover, using concentrated PV increased the system efficiency from 12 to 16% more than the non-concentrated one (Bicer and Dincer 2017). Hence, the mathematical study had the same results as the experiment (Ismail et al. 2019).

Furthermore, another study investigated the system’s performance with and without MPPT (Ganeshan et al. 2016). Their investigations revealed that although the system with direct coupling had an efficiency close to the system with MPPT, the capital cost of the system with MPPT was higher. Another study also mentioned that increasing MPPT efficiency increased hydrogen production (Rahim et al. 2015). Therefore, the PEM electrolyzer is suitable for PV/H_2_ due to its ability to deal with load fluctuation and its lowest cold startup property, among other types (Paul and Andrews 2008), as illustrated in Table 3.

Since early PV/H_2_ systems have low performances (2–6%) and high production costs (40 $/kg) (Gibson and Kelly 2008), PV panels and electrolyzer enhancements are employed to reduce costs with increasing production, which minimizes hydrogen production costs and enhances system performances. Based on this principle, a system’s efficiency was increased up to 12.4% instead of 6% by directly connecting PV with an electrolyzer (Gibson and Kelly 2010). Another study observed that hydrogen’s levelized cost (LCOH) ranged from 1.8 to 3.4 $/kg instead of 40 $/kg, which was mentioned before (Şevik 2022). This high efficiency was obtained when the output voltage from the PV panels was equal to or slightly higher than the required voltage for the electrolyzer. In yet another study, a PV/H2 with and without a battery system was compared to investigate their constant daily electric load consumption (Richards and Conibeer 2007). Investigations revealed that this system was more suitable as a stand-alone system in arid areas and areas with high elevations (Valdés et al. 2012). It was also reported that the tilted PV panels possessed higher efficiency during hydrogen production in a stand-alone system than horizontal panels (Tebibel 2017). However, the PV/H_2_ system without a battery requires fewer PV panels and involves low LCOH. Therefore, coupling PV panels with a water electrolyzer to produce the necessary hydrogen for fuel cells and provide electricity during the night or winter is a promising technique for the future (Lagorse et al. 2008).

Alternatively, purchasing electricity to operate the electrolyzer during solar radiation off-periods is economically viable due to the increase in working hours (Ferrari et al. 2016). The results demonstrated that since the LCOH values for grid/H_2_, grid+PV/H_2_, and PV/H_2_ were 5.5, 6.1, and 12.1 $/kg, respectively (Shaner et al. 2016; Grimm et al. 2020; Matute et al. 2022), the system’s cost could be recovered in 12 years. They also discovered that the enormous capital cost was mainly due to land costs and the amount used for PV panel construction. Therefore, remote areas with abundant solar radiation are considered more suitable locations for this type of plant. Since the optimal integration between PV panels and water electrolyzers is mandatory to provide high hydrogen production by increasing a system’s efficiency, monofacial and bifacial PV panels are used in PV/H_2_ systems to show their impact on efficiency under similar operating conditions (Privitera et al. 2020). Investigations revealed that the efficiency was up to 13.5% for bifacial PV panels rather than 11.55% for monofacial PV panels. Based on the bifacial PV, they also observed that hydrogen production increased from 3.7 to 4.2 g/h per square meter.

Remarkably, it was recently observed that an ultrahigh concentration PV/H_2_ system could improve a system’s efficiency to approximately 18–21% rather than 9.4% for conventional PV, with its hydrogen production ranging from 0.8 to 1.0 L/min per square meter for concentrated PV/H_2_ (Muhammad-Bashir et al. 2020). Although the battery increases the system capital cost, it reduces the electrolyzer’s size and enables hydrogen production at night, involving an LCOH around 6–7 €/kg (Gutiérrez-Martín et al. 2020; Zhang and Wei 2020; Puranen et al. 2021). Likewise, DCX converters use a high-efficiency DC voltage in PV/H_2_ to increase the system’s efficiency (Concha et al. 2021).

Since the performance of conventional PV panels increases by reducing their temperature, photovoltaic thermal (PVT) can introduce high-electricity output plus heat energy for several purposes (Li et al. 2022a) (Soliman and Hassan 2019). As a result, this electricity increases the hydrogen production from an electrolyzer, thereby reducing production costs. Furthermore, while air, water, and nanofluids as cooling fluids can reduce the PVT temperature, output fluid from PVT could be used for heating (Gado et al. 2022). Consequently, hydrogen production from coupling PV, PVT/air, and PVT/water with water electrolyzer reaches up to 8.19, 13.9, and 17.12 mL/min, respectively (Senthilraja et al. 2020), whereas PVT/nanofluids increase the power output by 47% compared with PVT/water (Sangeetha et al. 2021).

#### Multi-generation PV/H_2_ system

Electricity from PV panels can also produce hydrogen from electrolyzers in addition to other applications (e.g., space heating or cooling) (Erzen et al. 2020; Tukenmez et al. 2021; Şevik 2022). Therefore, these systems are currently attracting more attention due to their high performance compared to traditional PV/H_2_ systems. Table 4 summarizes the multi-generation techniques and their main specifications. Consequently, while the PV and PVT panels provide heat and electricity demand for buildings (Elghamry et al. 2019), their excess electricity drives the electrolyzer for hydrogen. Hydrogen notably drives fuel cells to provide heat, electricity, and sometimes drinkable water. Hence, in practice, using the Hoffmann voltammeter electrolyzer enables researchers to perform pretreatment for wastewater to reduce pollutants. Besides hydrogen production, the system can be used for ammonia production (Siddiqui and Dincer 2020). The results revealed that the system exergy efficiency reaches up to 55.5% and the produced ammonia is up to 1949.8 kmol, while hydrogen production is up to 5849.3 kmol.

**Table 4:**
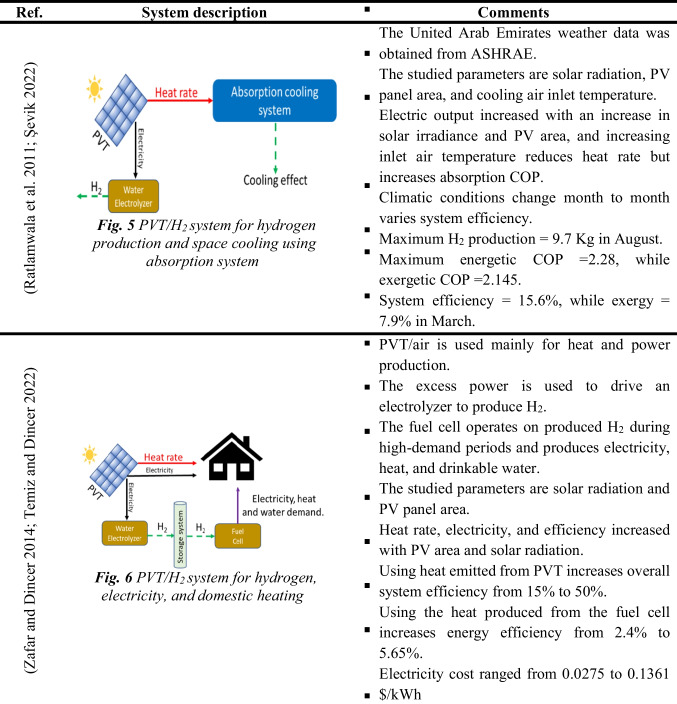

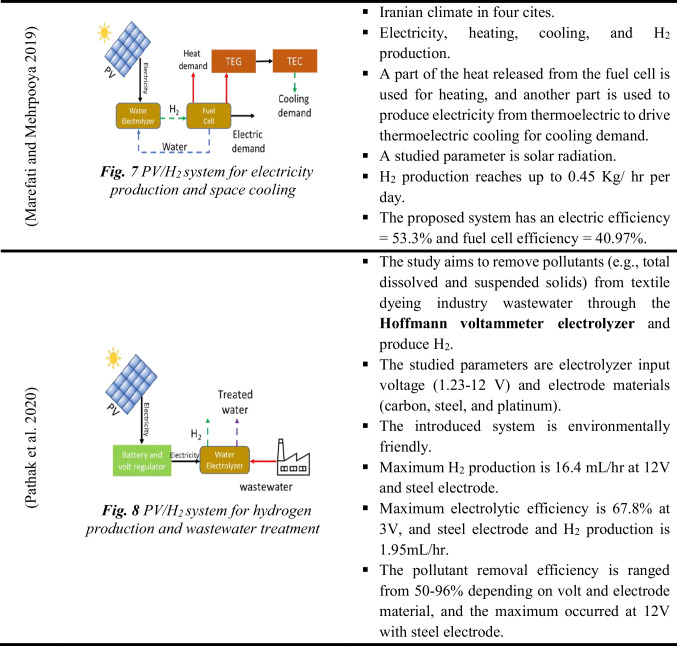

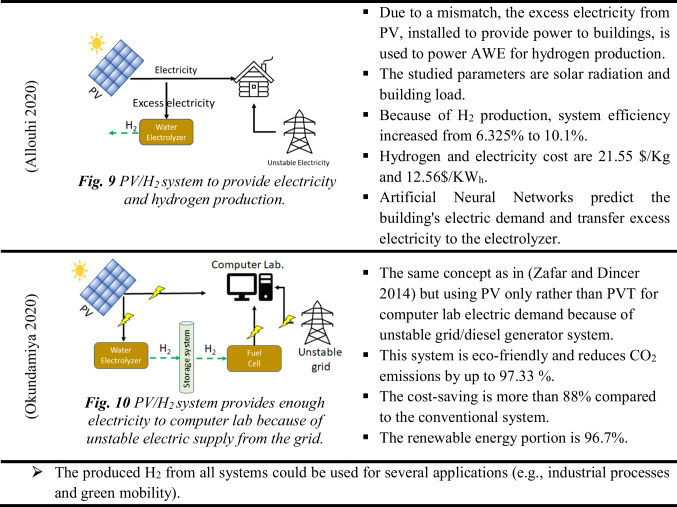
The multi-generation hydrogen system

• The produced H2 from all systems could be used for several applications (e.g., industrial processes

and green mobility).

The multi-generation system can use other energy sources besides solar (Hassan et al. 2022). For example, integrating PV panels with a mini-hydro plant for electricity production to drive an electrolyzer increases hydrogen production from 50,554 to 62,568 Nm^3^/year (Pereira et al. 2017). As a result, while electricity from the PV was 22.5% of the total produced electricity, the remaining part was from a mini-hydro plant. Furthermore, integrating a flat plate collector with the ocean thermal energy conversion (OTEC) cycle to produce electricity for the water electrolyzer has also been introduced (Yilmaz et al. 2018). Investigations proved that while the exergonic efficiency ranged from 22 to 36.49%, hydrogen production was about 1.2 Kg/h.

#### PV/H_2_ case studies

Several case studies have been conducted to estimate and analyze hydrogen production amounts, including the cost of producing 1 kg of hydrogen using PV panels under different climatic conditions (e.g., Algeria and Morocco). For example, a numerical study was conducted in the United States to show the ability of the PV/H_2_ system to supply the first two fuel cell buses with hydrogen (Vidueira et al. 2003). Their investigations revealed that the system could provide the buses with enough hydrogen to operate all day without emission. In other studies, while the potential for hydrogen production in southern Algerian regions proved better than in the northern areas by 45% under the same conditions (Mokhtara et al. 2020; Khelfaoui et al. 2020), other regions of Algeria recorded a higher hydrogen production than North African countries (Saadi et al. 2016). For instance, under the Moroccan climate, LCOH ranged from 4.64 to 5.79 $/kg when the electricity cost from PV panels was 0.077–0.099 $/kWh (Touili et al. 2018). Hence, a study compared fixed PV, PV with a tracking system, and a Stirling dish for H_2_ production in some regions (Touili et al. 2020). Their results confirmed that while the LCOH during fixed PV was the lowest, reaching up to 5.8 $/kg, the Stirling dish/H_2_ had the highest efficiency. However, this cost was lower than 5.96 $/kg in Southern Spain, 6.51 $/kg for South Africa, and 6.6 $/kg for the United States (Koleva et al. 2021). Another study used 400-watt PV panels in Cotonou, Benin. The hydrogen production was 115 L/day for 1.09 €/m^3^ based on their climatic conditions (Fopah-Lele et al. 2021).

### CSP/H_2_ system

**Fig. 5:**
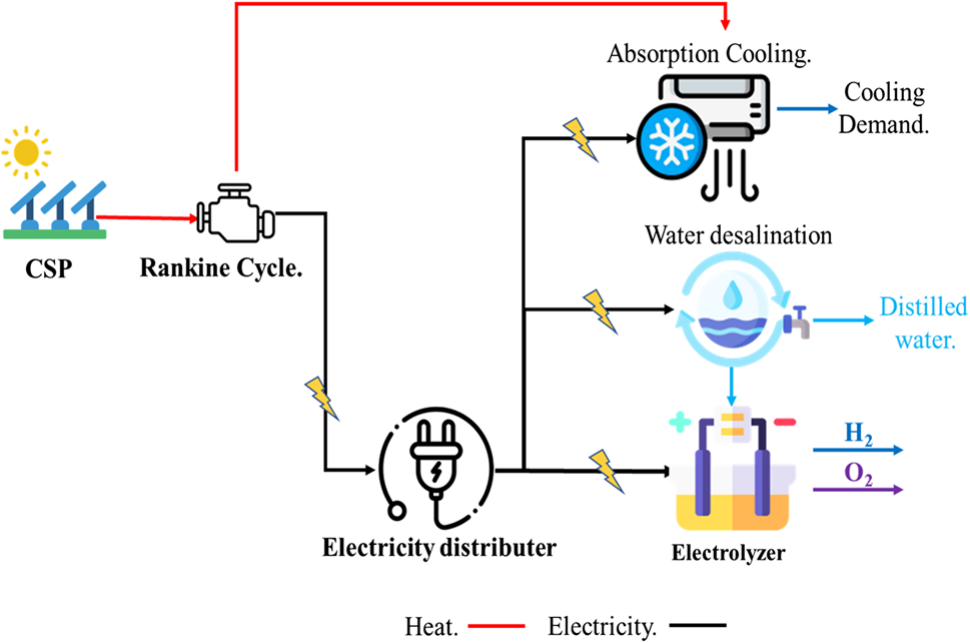
The schematic diagram of the CSP/H_2_ multi-generation system (Chadegani et al. 2018)

As previously stated, the CSP/H_2_ system generates electricity and heat to operate the electrolyzer and other methods. In such a system, solar radiation is collected and concentrated using a solar collector (e.g., flat plate collector (FPC) and parabolic dish collector (PDC)). While the generated heat is employed to drive the power cycle for electricity production, another portion is used to produce steam, like in the case of SOE, or to power absorption cooling cycles, as presented in Fig. 5. The power cycles used in this system are organic Rankine cycle (ORC) and Brayton cycle. Mainly, ORC attracts more attention because it works with low-grade heat. Table 5 summarizes the main specifications for a CSP/H_2_ system.

**Table 5:** CSP/H_2_ system specifications and performance

Ref.	Collector type	Cycle	Heat storage system	Auxiliary heater	Electrolyzer type	H_2_ production	H_2_ efficiency	Exergy efficiency	Notes
(Sun et al. 2012)	FPC	CO_2_Rankine	✓	✓	AWE	2.1 L/s	5.1 %	12.38%	–
(Ozturk and Dincer 2013)	PDC	Steam Rankine	✓	–	SOE	–	21.3%	14.31	Sub-systems: power, heating, and cooling
(Sanz-Bermejo et al. 2014a)	LFC	–	✓	✓	SOE	500 Kg/day	43.1%	–	Grid for electricity and LFC for heat
(Koumi Ngoh et al. 2014)	PTC	–	–	–	SOE	0.064 Kg/s	–	–	Electricity from PV panels
(Sanz-Bermejo et al. 2014b)	Heliostat field	Steam Rankine	–	–	SOE	3100 m^3^/h	5.8%	–	Electricity consumption is 3.3KW_h_/Nm^3^ of H_2_
(Akikur et al. 2014)	PTC	–	✓	✓	SOE	0.00241 Kg/s	20%	–	Electricity from PV panels
(Houaijia et al. 2015)	Heliostat field	Steam Rankine	✓	–	SOE	680.4 Kg/h	18%	–	Production under steady-state conditions
(Al Zahrani and Dincer 2016)	Heliostat field	Brayton	✓	–	SOE	20.6 Kg/h	12.7%	13.9%	–
(Balta et al. 2016)	Heliostat field	Rankine+ORC+Brayton	–	–	SOE	0.057 Kg/s	24.79%	22.36%	Electricity consumption is 1.98 KW_h_ for 0.057 Kg/s of H_2_
(Naseri et al. 2017)	FPC	CO_2_ Rankine+Natural gas turbine	✓	–	AWE	1.309 L/s	–	–	Electricity production from Stirling engine.
									Distilled water production is 0.964L/s
(Moradi Nafchi et al. 2018)	Heliostat field	Steam Rankine	✓	–	PEM	0.8 Kg/h	20.1% ^a^	41.25%^a^	High-temperature PEM electrolyzer
(Atiz et al. 2019)	ETC	Isobutene Rankine	✓	–	–	3.024 Kg/day	5.92% ^a^	18.21% ^a^	–
(Qureshy and Dincer 2020)	Heliostat field	Steam Rankine	✓	–	–	–	28.9%	31.1%	–
(Atiz 2020)	PTC	n-butane Rankine	–	–	PEM	2.758 Kg/day	5.67%	7.49%	Geothermal energy is used before the solar collector to increase inlet temperature
(Mastropasqua et al. 2020)	PDC	Brayton	–	–	SOE	150 Kg/day	30%^b^	–	Multi PDC with a micro gas turbine is each dish’s focal point
(He et al. 2020)	PTC	Brayton	✓	–	SOE	4.32 Kg/h	21.5%	22.5%	12 h running time
(Delpisheh et al. 2021)	PTC	n-octane Rankine	–	–	PEM	15.6 Kg/h	12.29 %	5.11%	Distilled water production is 1.76 Kg/s
(Hosseini and Butler 2021)	CPVT	ORC	–	–	AWE	0.1587 Kg/day	–	–	Electricity produced increased up to 30% by using ORC+CPVT rather than CPV
(Khouya 2021)	Heliostat field	Steam Rankine + CPVT	✓	–	PEM	628Kg/h	–	–	Hydrogen production cost is 6.86 $/Kg
(Seyyedi et al. 2022)	PTC	Rankine	–	–	PEM	2.5Kg/h	–	–	Hydrogen production cost is 6 $/Kg
(Puig-samper et al. 2022)	PTC	Steam Rankine	✓	–	SOE	5–60 Ton/month	–	–	Hydrogen carbon footprint (1.85kg_CO2_/kg_H2_)

H_2_ efficiency is solar to hydrogen efficiency, defined as the ratio between power produced from hydrogen and solar energy input rate

^a^Hydrogen and electricity efficiency

^b^Solar to hydrogen efficiency when SOE efficiency is 80% and hydrogen cost is 5.9–9.1 €/ kg

A CSP/H_2_ system can also be used for hydrogen production, electricity production, heating, cooling, and freshwater supply (Fig. 5). These multi-generation techniques enhance the total system’s efficiency (Chadegani et al. 2018). To this end, a study reported that the energy and exergy efficiency of a CSP/H_2_ system could vary from 33.52 to 71.6% and 20.7 to 36%, respectively (Delpisheh et al. 2021; Gill et al. 2021). It was also reported that a CSP/H_2_ multi-generation system’s overall efficiency increases when the solar radiation improves, lowering the electrolyzer’s working temperature and growing its current density (Chen et al. 2020). Hence, these systems could reduce CO_2_ emissions.

Notably, CSP/H_2_-based multi-generation systems also employ other energy sources (e.g., geothermal energy) to enhance the system’s efficiency and better use of available energy sources in regions (Sen et al. 2021; Temiz and Dincer 2021). To this end, a study reported that while LCOH reached up to 2.84 $/kg using a multi-generation system, the levelized cost of electricity (LCOE) reached 0.03 $/kWh. In yet another study, a Stirling engine was installed at the focus point of a solar concentrator to produce electricity directly, which regulated the water electrolyzer (Marefati et al. 2019; Zayed et al. 2021). Based on these facts, the amount of hydrogen produced using the PV and CSP/Stirling systems was compared (Lahoussine Ouali et al. 2020). Investigations revealed that the hydrogen produced was 302.2 kg for CSP/Stirling systems and 267.8 kg for PV/H_2_.

### The wind/H2 system

**Fig. 6:**
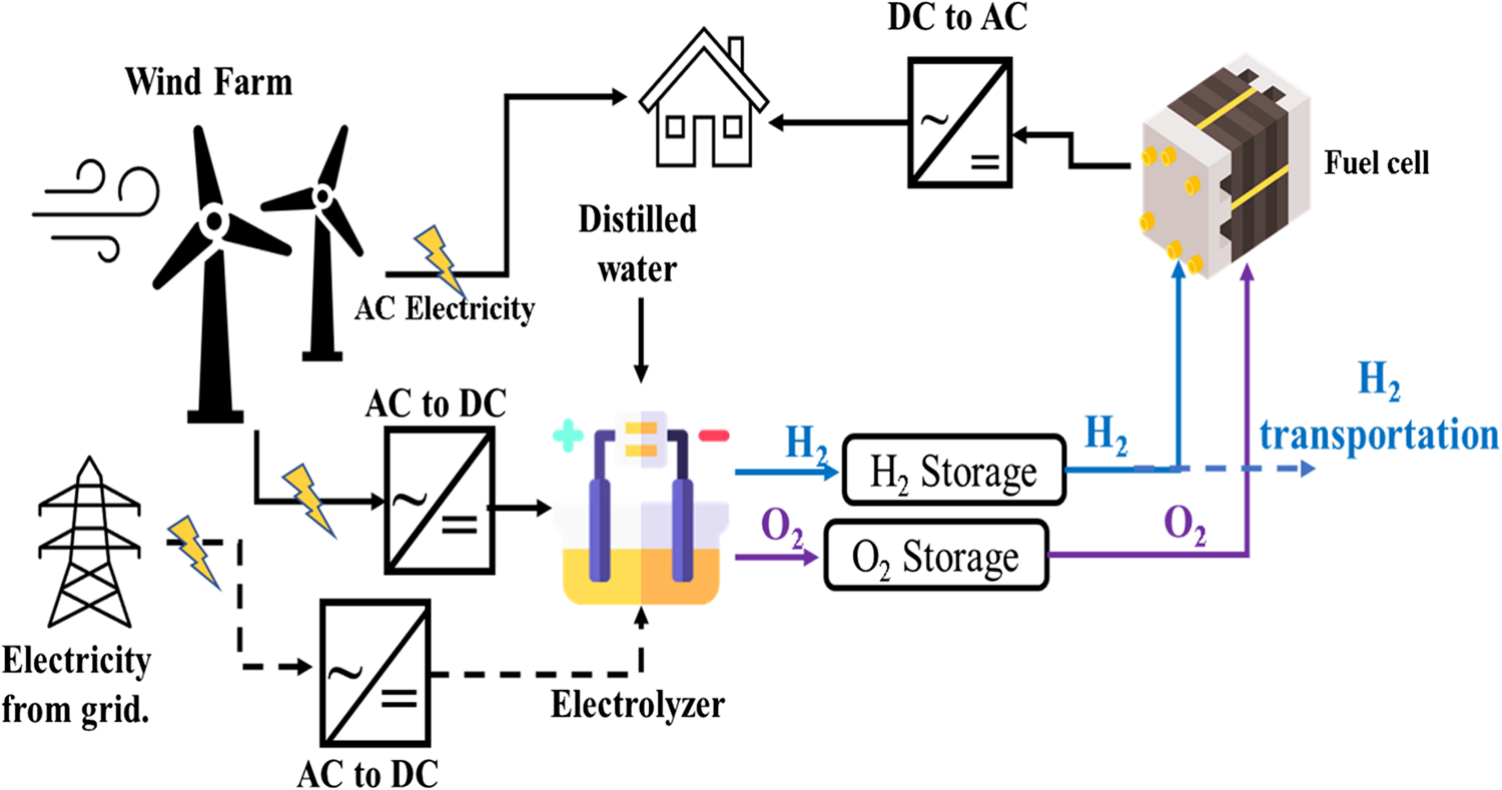
The schematic diagram for the Wind /H_2_ system for hydrogen and electricity production

Due to wind’s unpredictable and intermittent nature, wind turbines’ electricity fluctuates throughout their operational period. Therefore, a time exists in the operation period when the produced electricity is higher or lower than the required electric demand. During this period, excess electricity must be stored. To this end, the wind/H_2_ system has been proposed as a solution for long-term energy storage of electricity in the form of hydrogen gas because this gas converts to electricity again during low production periods, as demonstrated in Fig. 6. Subsequently, the electrolyzer can be operated based on the accumulated electricity from the wind turbine, or using the whole electricity from hydrogen production, the produced hydrogen can also be sold instead of converted to electricity (Carton and Olabi 2010; Xiao et al. 2020). Therefore, coupling a wind turbine with a water electrolyzer and a fuel cell provides a sustainable and clean energy solution (Dabar et al. 2022). Furthermore, combining wind energy and water electrolyzer also increases the performance of wind turbines and the produced hydrogen used as a backup unit (Liu et al. 2021).

A wind/H_2_ system is constructed by coupling a wind turbine generator with an AC/DC converter before attaching them to a water electrolyzer (Nadaleti et al. 2019). In this system, produced hydrogen is employed for several applications according to the following scenarios: The first is the wind/H_2_ grid–independent scenario, where the water electrolyzer is directly coupled with wind energy through a power-conditioning system. This scenario is more suitable in remote areas, where most wind farms are installed (Luo et al. 2022). The second scenario is the wind/H_2_ grid–assisted system, where the electricity from the grid near the wind farm is used to help the wind turbine produce hydrogen due to wind intermittency. While the third scenario involves wind energy that drives the electrolyzer during excess production and provides electricity to the grid, the fourth is similar to the previous one and contains a storage system and fuel cell for electricity production (Zhou and Francois 2009; Geovanni et al. 2010). Finally, the last one has a storage system and serves two purposes: (I) to power the fuel cell and produce electricity and (II) to transport part of the stored hydrogen for other purposes (Sherif et al. 2005). Any excess hydrogen produced can then be used in fueling stations and methanation processes. Fig. 6 shows the essential components of all scenarios.

Based on the principles above, a study coupled a horizontal axis wind turbine with AWE and a fuel cell to produce a constant electric supply in the Aegean islands (Iqbal 2003; Ntziachristos et al. 2005). Their results showed that the system’s overall efficiency reached 60% due to this configuration. For the first time, another study also showed that the wind/H_2_ system could provide ten households with electricity for 2–3 days (Ulleberg et al. 2010). However, the electricity cost of this system is incomparable to that of conventional ones. Nevertheless, cost implications are proposed to be reduced in the future due to the taxes on CO_2_ emissions and an increase in fossil fuel costs. A primary concern about the wind farm still persists: the power fluctuation changes based on wind speed. To this end, a novel switching strategy with a chopper circuit for each electrolyzer has been used to regulate the input electricity to the electrolyzer (Muyeen et al. 2011). The results showed that each electrolyzer worked full load, increasing its lifespan and efficiency. Another study that used a vertical axis wind turbine in the wind/H_2_ system has also reported acceptable performance (Demirdelen et al. 2020).

Notably, the wind/H_2_ system has been investigated numerically using four electrolyzer models (Sarrias-Mena et al. 2015). The investigations revealed that all models performed similarly under different wind speed conditions. Subsequently, the system’s performance was enhanced by combining more than one control method (Fang and Liang 2019; Qiu et al. 2019; Saenz-Aguirre et al. 2020). Other studies have also investigated the performance of a new wind/H_2_ system strategy (Grüger et al. 2019). The results showed that the production cost was reduced by 9%, from 13.28 to 11.52 €/kg. Similarly, the wind/H_2_ system was investigated under different capacity factors in Kuwait (Sedaghat et al. 2020). The results illustrated the system could produce 0.01 Kg/h when consuming 628.4 W from a 2-kW wind turbine (Shen et al. 2021).

Remarkably, studies have also proven that hydrogen production costs from wind/H_2_ systems vary from one place to another according to the amount and price of electricity produced from wind turbines and electrolyzer costs. For example, in Afghanistan, while the LCOH ranged from 2.118 to 2.261 $/kg, the LCOE was 0.063–0.079 $/kWh (Rezaei et al. 2020). However, in Yazd City, Iran, the LCOE and LCOH implications were 0.068–0.115 $/kWh and 2.1008–3.5602 $/kg, respectively (Almutairi et al. 2021). In contrast, while the LCOE and LCOH implications reduced to 0.0325–0.0755 $/kWh and 1.375–1.59 $/kg in Lutak City, Iran, due to the high wind speed conditions in this region (Rezaei et al. 2021), the LCOH was 3.1 and 4.02 $/kg in Turkey and Pakistan, respectively (Genç et al. 2012; Iqbal et al. 2019). Moreover, while the LCOH in South Africa was between 6.34 and 8.97 $/kg (Ayodele et al. 2021), it was 7.3 $/kg in Germany (Herwartz et al. 2021).

Hydrogen can be physically stored as a compressed gas in a storage tank under high pressure for transportation. However, the main concern with hydrogen storage is the leakage of compressed gas (Li et al. 2022b), as experiencing a high-pressure hydrogen leak can result in an explosion, leading to significant injuries and property damage. Accordingly, a previous study (Nasser et al. 2022a) established that the storage system raises hydrogen production costs due to the increased capital cost of system components. The results revealed that the production cost increased by 50% when a storage system was used. Based on this limitation, installing offshore wind turbines during hydrogen production from the water electrolyzer has been investigated under different scenarios (Franco et al. 2021). Investigations revealed that the LCOH implication was 5.35 €/kg when transporting hydrogen to the shore by pipeline, which was lower than gas liquefaction. This cost could be reduced to 2.17 €/kg if the European Union supported the hydrogen production project. In another study, the hydrogen produced from the wind/H_2_ system was coupled with a methane production unit (Ishaq and Dincer 2020). This combination generated 3.4 g/s and 52.25 g/s of hydrogen and methane, respectively.

Moreover, the results showed that while carbon dioxide emissions were reduced by 2999 tons/year, the system efficiency and exergy were 42.3% and 40.5%, respectively. As a result, the wind/H_2_ system had six times higher energy and exergy efficiencies than the OTEC/H_2_ system (Ishaq and Dincer 2020). Finally, wind and CPVT energy sources were compared on the basis of hydrogen production in Morocco (Khouya 2020). Investigations revealed that the LCOH ranged from 2.36 to 2.66 $/kg for wind energy to 3.17–4.54 $/kg for CPVT. This comparison was convenient because while wind turbines worked day and night, solar worked only during the day.

### Hybrid solar and wind hydrogen production system

The potential to find a stand-alone system to produce electricity in remote areas gets more attention day by day. Producing hydrogen from solar and wind energy is stored for electricity production via a fuel cell in case of excess electricity or selling hydrogen directly to the market (Bernal-Agustín and Dufo-López 2010; Nasser et al. 2022b). The main drawback of using wind and solar separately is the high hydrogen production cost compared to other energy sources, as mentioned above. Therefore, combining wind and solar energy to create a hybrid hydrogen production system (WS/H_2_) might provide a cost-reduction solution (Nasser et al. 2022a). Moreover, this system offers continuous production because it depends on two energy sources to avoid intermittency periods.

**Fig. 7:**
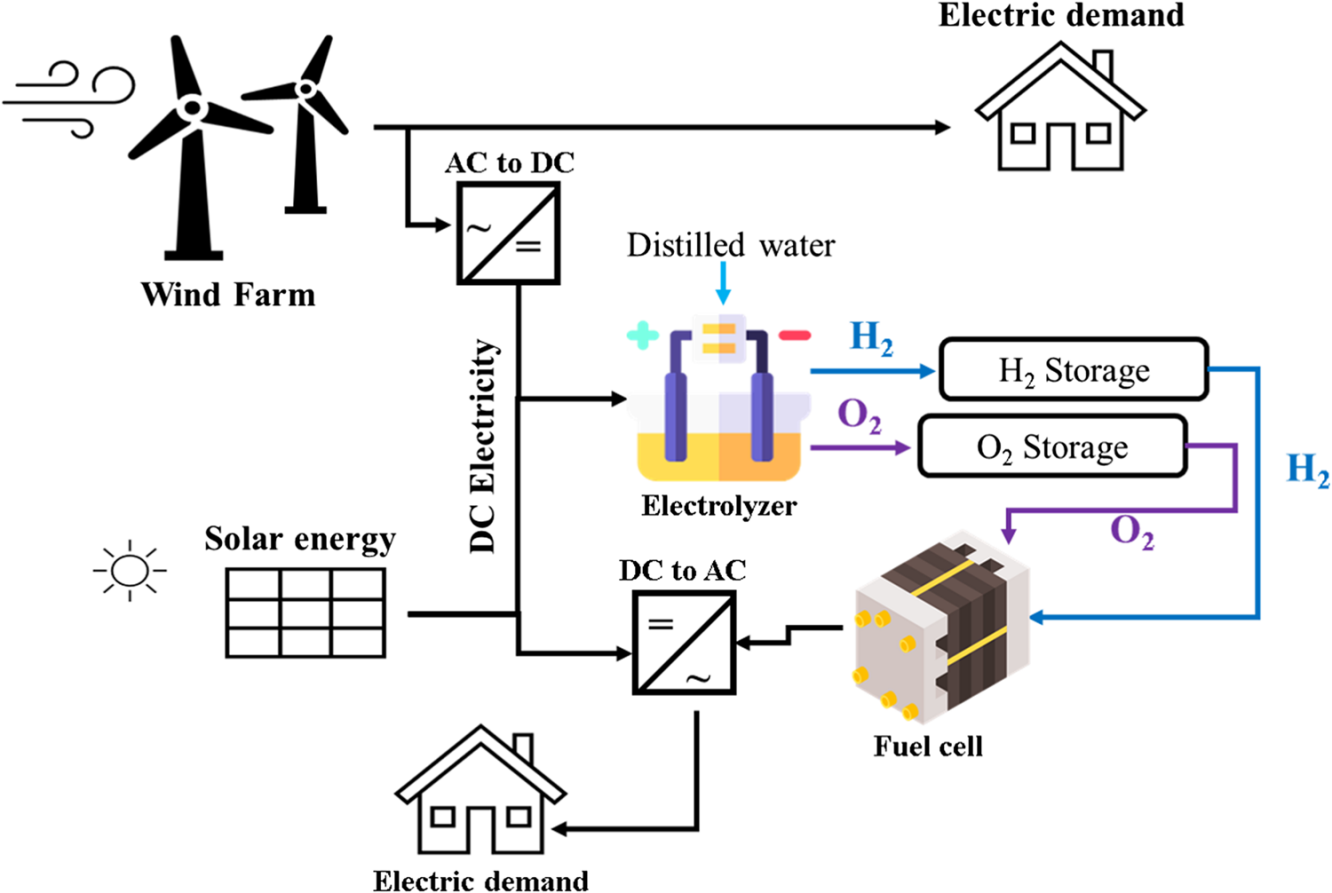
The schematic diagram for a hybrid hydrogen production system

The basic concept of the WS/H_2_ system is illustrated in (Nasser et al. 2022a; Babatunde et al. 2022) and shown in Fig. 7. This system offers excellent potential in electricity production compared to the traditional one because of the combination of solar and wind energy. Additionally, selling hydrogen at 10 €/kg is economically viable in areas with high wind speed. The hybrid can be created by combining PV/H_2_ and wind/H_2_ systems (Akyuz et al. 2012; Babatunde et al. 2022). The results demonstrated that the efficiency of PV/H_2_ and wind/H_2_ is 7.9–8.5% and 5–14%, respectively, with hydrogen production of 30.4 kg between April and July.

The hydrogen production from the hybrid system is more than wind/H_2_ and PV/H_2_ by 26.2% and 127%, respectively (Khalilnejad and Riahy 2014; Huang et al. 2015). Furthermore, wind turbine contribution is more than PV panels because it works all day and PV panels work at noon only. The thermal efficiency of combining a wind farm with CPVT is better than combining it with CSP (Cai et al. 2020).

The efficiency of hydrogen production increased when using the hybrid system by two factors: (I) an increase in electricity input to the electrolyzer and (II) an increase in the water temperature by solar energy before entering the electrolyzer (Huang et al. 2016). Hydrogen production reaches up to 0.51 Kg/h, and it can be used for refueling stations for hydrogen vehicles. The cost reached 12.3 €/kg when the electricity cost 10 c€/KWh (Bernal-Agustín and Dufo-López 2010). In addition, when a battery system is installed with WS/H_2_ system, the number of electrolyzer stops is reduced, and working hours, efficiency, and lifetime boost (Ursúa et al. 2016). The hybrid system’s utilization factor is higher than the single system (Papadopoulos et al. 2018).

Some studies are done to investigate the uses of this system in real life: for electricity production for 150m^2^ houses (Devrim and Bilir 2016), providing hydrogen for hydrogen vehicles (Rezaei et al. 2019; van der Roest et al. 2020) and ammonia, and urea production (Armijo and Philibert 2020; Ishaq et al. 2021). The results proved that the proposed system could provide a house in Turkey with the required electricity around the year except for November. Similarly, the system produces 91 Kg/day of hydrogen, providing 91 cars with energy per week in Iran. In Chile and Argentina, ammonia is manufactured directly from the system with 500 $/ton when the hydrogen costs 2 $/Kg.

**Fig. 8:**
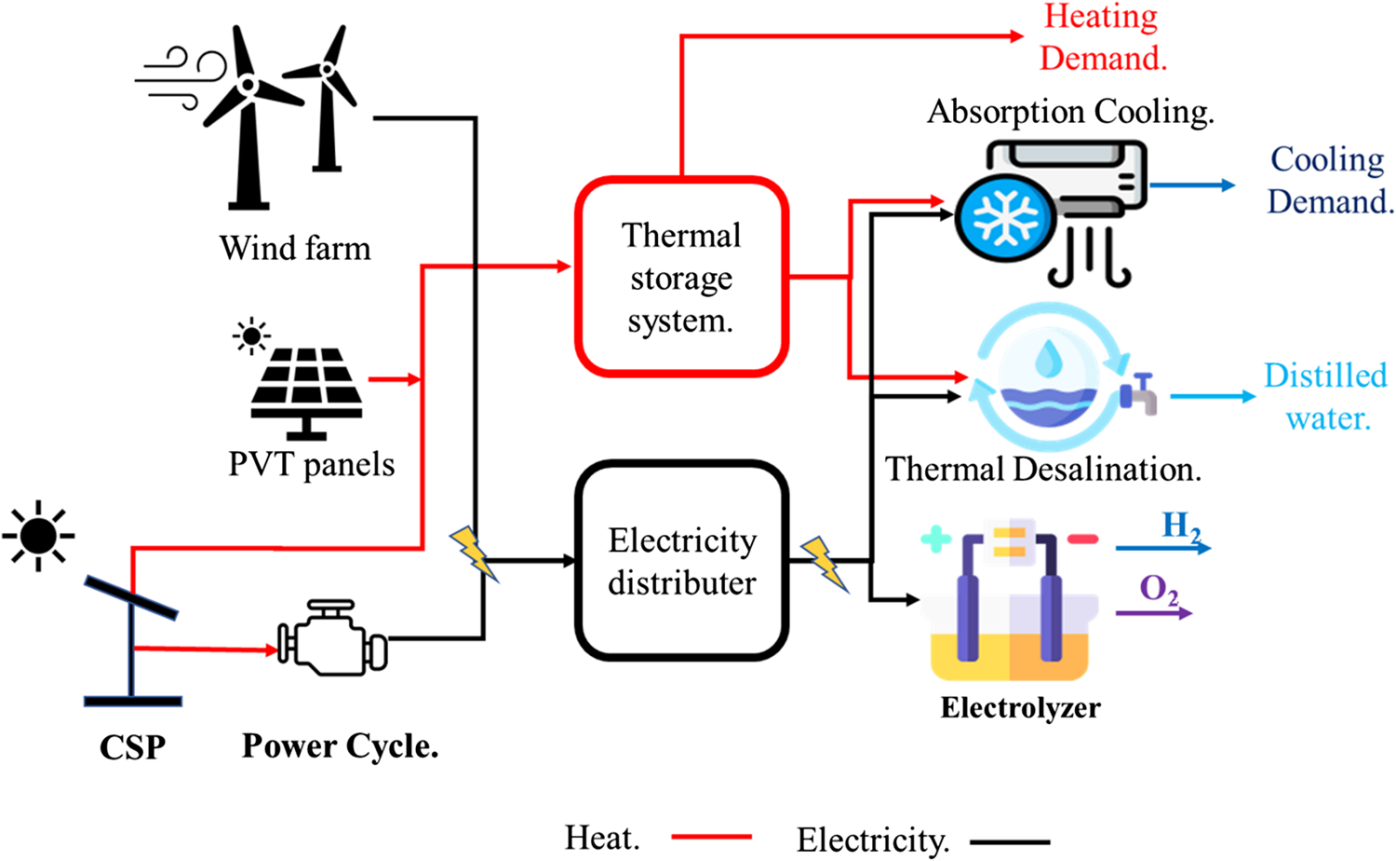
The schematic diagram for a multi-generation hybrid system for hydrogen production, space cooling, heating, and desalination (Sezer et al. 2019)

In Canada, the hybrid system is designed for hydrogen and urea with 518.4 kmol/day and 86.4 kmol/day, respectively (Ishaq et al. 2021). This hybrid system can provide 540 hydrogen-electric vehicles with the required hydrogen at LCOH equal to 8.7 €/kg, which is lower than the end-user cost (10 €/kg) in the Netherlands (van der Roest et al. 2020). This LCOH can be reduced by 20–26% when considering the avoiding cost of CO_2_ emissions, which is about 3600 tons per year. Additionally, the introduced system provides distilled water to the electrolyzer by the reverse osmosis system powered by renewable energy. Finally, the hybrid system is used not only for hydrogen production but also for space cooling, heating, and desalination, as shown in Fig. 8. The energy and exergy efficiency of the whole system is 61.34% and 47.8%, respectively, with hydrogen production of 239 Kg/h (Sezer et al. 2019). The dissipated heat from a fuel cell is also used to operate a Stirling engine for electricity production (Wang et al. 2021). The results showed that the Stirling engine produces about 1033.7 W representing 9.85% of the output power.

## Economic assessment of green hydrogen production

**Fig. 9:**
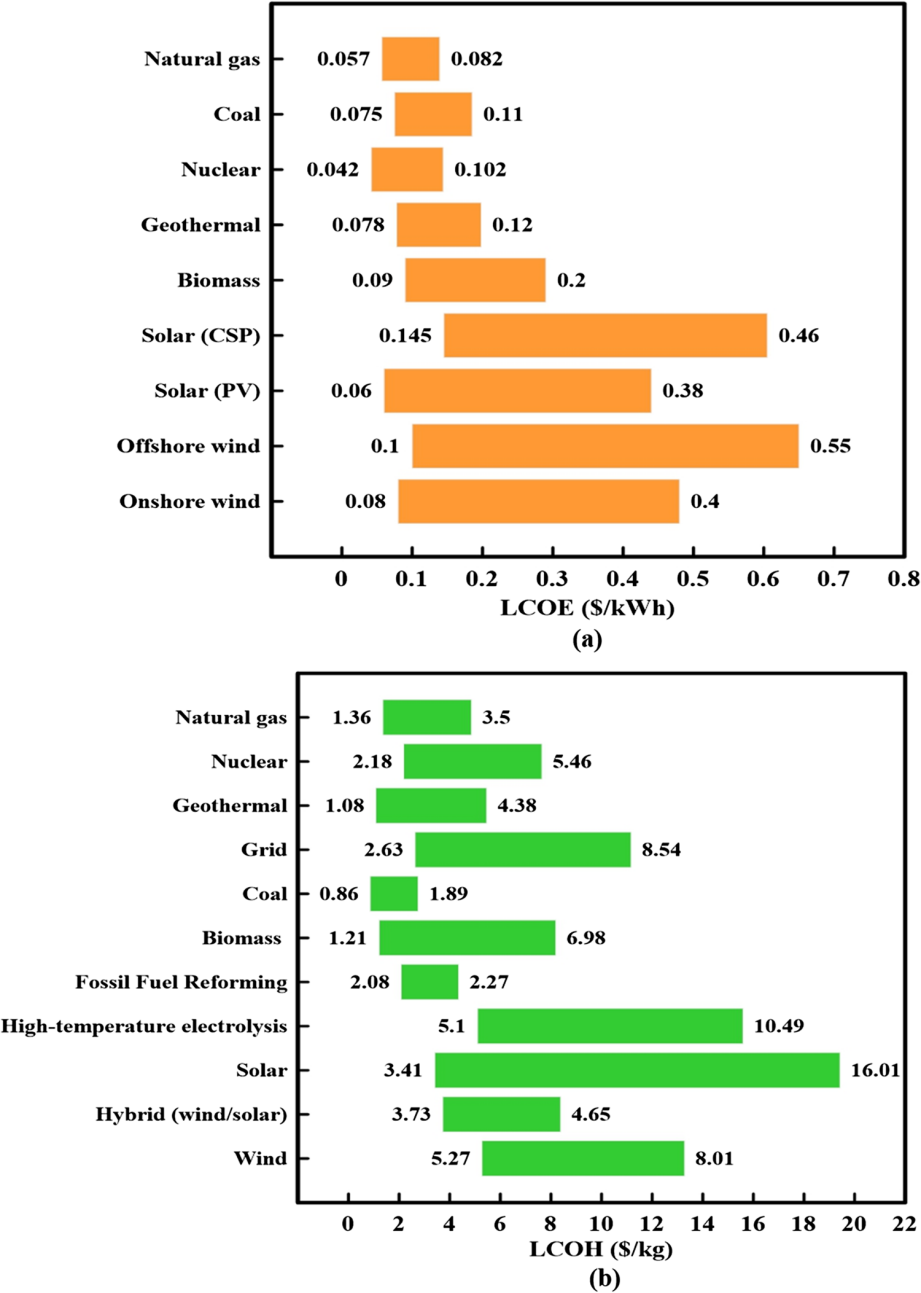
The range of (**a**) LCOE and (**b**) LCOH from different energy sources (El-Emam and Özcan 2019; IEA 2020; Razi and Dincer 2020; Shen et al. 2020; Wu et al. 2021)

Electricity plays a vital role in hydrogen production because it is the primary input to electrolyzers that controls hydrogen production costs. Moreover, several factors such as the lifetime, land cost, and construction period influence electricity production’s pricing from renewable energy. Fig. 9a demonstrates the LCOE implications for different energy sources (renewable and nonrenewable) (Shen et al. 2020). This figure shows that although traditional power sources like coal and nuclear have the lowest LCOE, they negatively affect the environment due to green gas emissions. In contrast, while renewable energy sources have higher LCOEs than traditional ones, they are preferred nowadays because the world is trying to convert to zero emission power generation, proposing that its cost will be reduced.

When calculating LCOH, electricity cost directly affects the results (Nasser et al. 2022a, 2022b). Fig. 9b illustrates the LCOH for different techniques (El-Emam and Özcan 2019; Razi and Dincer 2020). As shown in this figure, the production methods that depend on conventional energy sources had lower LCOH than green methods. Notably, although these methods were expensive, they have attracted more attention for being used in constructing a sustainable society. For example, while the LCOH produced from solar and wind energies varied from 3.41 to 16.01 $/kg and 5.27 to 8.01 $/kg, respectively, the LCOH of a hybrid system lay in the midway of the solar and wind.

**Fig. 10:**
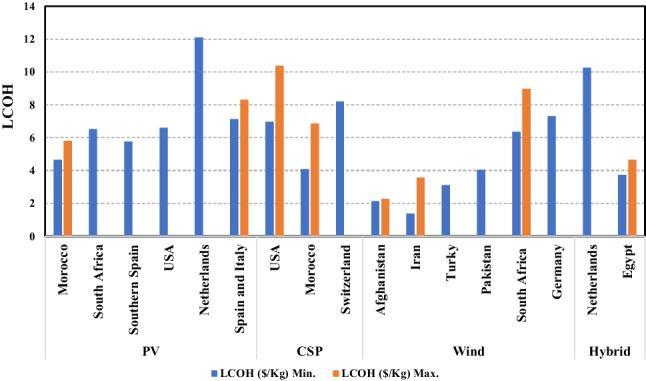
LCOH according to the country of production (data collected from the review)

Climatic conditions also play a crucial role in producing electricity and hydrogen from solar and wind energy due to the dependence of these sources on climate. As a result, the production of each system mentioned in this study is influenced by location changes from one place to another. Fig. 10 illustrates the LCOH gathered from this review for the understudied systems. This figure demonstrates that although the LCOH varied by the country for similar designs, the average cost of hydrogen production for wind energy was lower than that for solar because it worked day and night.

Studies have also reported that the electrolyzer type coupled with similar energy sources changes the LCOH. For example, LCOH from wind energy associated with PEM varies from 5 to 9.37 $/kg (Olateju et al. 2014, 2016; El-Emam and Özcan 2019). However, for AWE, the LCOH ranged from 7.47 to 7.6 $/kg (Greiner et al. 2007). In other studies, the LCOH when SOE is used varies from 6 to 9.2 $/kg and should decline to 2 $/kg by 2050 (Mastropasqua et al. 2020; Khatiwada et al. 2022). Thus, to sum up, the two main parameters that influence the LCOH from renewable sources are (I) weather conditions (e.g., solar radiation and wind speed) and (II) electrolyzer type.

### Economic challenges

Green hydrogen production is expensive and may remain so without government support and action. This fact is because in developing countries with a vast supply of natural resources for power generation, minimized hydrogen production costs are observed, accounting for distance and demand. Even in areas with abundant renewable energy resources, electricity represents a massive part of the manufacturing expenses, with electrolyzers and other expenditures being insignificant.

Therefore, authorities must enhance their energy budget to encourage green hydrogen. For example, the savings from reducing fossil fuel subsidies could be used to fund green hydrogen fuel. However, increasing production would need developing hydrogen infrastructure, a massive effort that requires a solid plan and political backing. Therefore, the authorities should collaborate with recognized firms in creating green hydrogen infrastructure to develop a strategic plan for green hydrogen’s success in the market and the expansion of such infrastructure (Agaton et al. 2022).

## Conclusion

A clean energy carrier, hydrogen, is expected to significantly influence this millennium by offering an ecologically friendly choice to meet the world’s rising energy needs. Moreover, since current research has focused on developing green methods for shifting toward a viable and cost-effective hydrogen economy to race with fossil fuel hydrogen generation, this study focused on hydrogen production from the green path using wind and solar energy. The conclusions drawn from this review work are as follows:

- Hydrogen is an alternate energy carrier that can be stored and transferred and has a high calorific value, making it suited to replace fossil fuels.
- Green production attracts more attention due to its ability to produce hydrogen with zero carbon emissions. However, coupling the energy source with a water electrolyzer is a better production technique.
- The electrolyzers can be divided into low-temperature electrolyzers (PEM, AWE, and AEM) and high-temperature electrolyzer (SOE). The low-temperature electrolyzers do not need any heat source and only require an electric source. However, a heat source helps to convert water to steam in SOE, including a power source for water decomposition.
- PEM electrolyzers are the most suitable to be attached to a green production system due to their low-start period. As a result, they can deal with load fluctuations. However, the SOE type is also suitable for dealing with the CSP/H_2_ system due to the high temperatures produced by CSP.
- PV/H_2_ and wind/H_2_ systems do not need any power cycle for electricity production, so they need minimum maintenance attention and are suitable for installation in arid areas. In contrast, CSP/H_2_ requires a power cycle, so maintenance cost must be considered.
- Although green hydrogen production systems are used for multi-generation purposes such as heating, cooling, and water desalination besides hydrogen production, the system’s performance depends on several factors, such as climatic conditions, tracking systems, control systems, and the electrolyzer type.
- The LCOH of WS/H_2_ and solar/H_2_ is relatively similar, but the LCOH of wind/H_2_ is higher. Therefore, although a hydrogen compression system raises the LCOH due to the increased capital cost, government support should be able to reduce this LCOH.
- The primary goal of commercializing a green hydrogen production system is to reduce the capital investment of its components and improve the components’ efficiency. These enhancements will enable green hydrogen to compete with other production methods in the global market.

## Data Availability

Not applicable.
